# Buffalo Hospital of the Sisters of Charity

**Published:** 1849-09

**Authors:** 


*Buffalo Hospital of the Sisters of Charity.*—An addition to this hospipal, which will double its capacity, is rapidly progressing. When completed, the institution will be able to accommodate from 250 to 300 patients. About 120 cases of cholera have been treated at this hospital during the late epidemic. A semi-annual report, now due, of the institution, may be expected in our next number.

